# Differential responses of Miocene rodent metacommunities to global climatic changes were mediated by environmental context

**DOI:** 10.1038/s41598-018-20900-5

**Published:** 2018-02-06

**Authors:** Fernando Blanco, Ana Rosa Gómez Cano, Juan L. Cantalapiedra, M. Soledad Domingo, Laura Domingo, Iris Menéndez, Lawrence J. Flynn, Manuel Hernández Fernández

**Affiliations:** 1 Departamento de Geodinámica, Estratigrafía y Paleontología Facultad de Ciencias Geológicas, Universidad Complutense de Madrid. C/José Antonio Novais 12, 28040 Madrid, Spain; 2 Transmitting Science. C/Gardenia 2, Piera, 08784 Spain; 3 Institut Català de Paleontologia Miquel Crusafont. Edifici ICP, Campus de la UAB s/n, 08193 Cerdanyola del Vallès, Spain; 4 Museum für Naturkunde, Leibniz-Institut für Evolutions und Biodiversitätsforschung, Invalidenstraße 43, 10115 Berlin, Germany; 5 Departamento de Ecología Evolutiva, Estación Biológica de Doñana (CSIC). C/Américo Vespucio 26, 41092 Seville, Spain; 6 Earth and Planetary Sciences Department, University of California Santa Cruz. 1156 High Street, CA 95064 Santa Cruz, USA; 7 Departamento de Cambio Medioambiental, Instituto de Geociencias (UCM, CSIC). C/ José Antonio Novais 12, 28040 Madrid, Spain; 8 Department of Human Evolutionary Biology, Harvard University. 11 Divinity Avenue, Cambridge, MA 02138 USA

## Abstract

The study of how long-term changes affect metacommunities is a relevant topic, that involves the evaluation of connections among biological assemblages across different spatio-temporal scales, in order to fully understand links between global changes and macroevolutionary patterns. We applied multivariate statistical analyses and diversity tests using a large data matrix of rodent fossil sites in order to analyse long-term faunal changes. Late Miocene rodent faunas from southwestern Europe were classified into metacommunities, presumably sharing ecological affinities, which followed temporal and environmental non-random assembly and disassembly patterns. Metacommunity dynamics of these faunas were driven by environmental changes associated with temperature variability, but there was also some influence from the aridity shifts described for this region during the late Miocene. Additionally, while variations in the structure of rodent assemblages were directly influenced by global climatic changes in the southern province, the northern sites showed a pattern of climatic influence mediated by diversity-dependent processes.

## Introduction

The traditional view of community ecology, where groups of species (communities) are temporally stable, closed and isolated from each other, has changed over the years^1–4^. Evidence shows that local communities are not only affected by local abiotic conditions and biotic interactions, but also by processes operating at regional scales such as speciation, extinction, immigration and emigration^4^. The metacommunity framework solves this issue, grouping local communities that are connected by the dispersal of one of the component species at least^1,5^. The application of the metacommunity concept is key to study the macroecological and macroevolutionary processes behind the deep time dynamics of communities^6–8^ and regional scale analyses allow us to evaluate macro-scale biotic and abiotic factors (faunal turnover, environmental change…) that affect them. Within this metacommunity framework, the study of assembly-disassembly processes, in which successive species losses and gains are considered the reflection of habitat changes usually linked to global climatic change, has gained relevance in the last years^8–10^. These studies have shown that nested patterns are frequent in the assembly and disassembly of metacommunities, since species endure or disappear in an orderly manner associated to the intensity of environmental disturbances. Nevertheless, although it is required to fully understand the link between biological assemblages and evolutionary biology^7,11,12^, the study of how long-term changes affect metacommunities remains incomplete.

We focus our work on the study of rodents because their communities are widespread, highly diverse and habitat-sensitive. Environmental disturbances commonly affect these communities, which recover from perturbations at relatively rapid rates. Moreover, the Iberoccitanian (southwestern Europe, including most of the Iberian Peninsula plus southern France, currently influenced by Mediterranean climate, see Supplementary Fig. 1 and the study area subsection in Methods section for its biogeographical relevance) fossil record of this group is characterised by a large amount of fossil remains due to their life cycle (r strategist) and the good preservation of their dentition, which are key for both systematic and paleoecological studies^13–17^. Finally, because of the high resolution of micromammalian fossil sites at large spatiotemporal scales, a great deal is already understood about the evolutionary dynamics of rodent faunas^6–9,13,18–20^. These particular features, make Miocene Iberoccitanian rodent metacommunities ideal to study the development of novel macroecological and macroevolutionary approaches (Supplementary Fig. 1). Additionally, their communities during the end of the Miocene (12 to 5 Ma) underwent major changes in a time interval of crucial climate shifts^21–24^, including the so-called Vallesian Crisis and the Messinian Salinity Crisis. The former has been related to the aridification of European ecosystems^22,25^, probably associated with gradual cooling during the Neogene and the initial development of ice sheets in the Arctic^26,27^, while the latter involved desiccation of the Mediterranean Sea and an associated sea level decrease in the Paratethys Sea^28^. In both cases, there is controversy about the effects on mammalian faunas^7,22,24,29–32^. Furthermore, both crises occurred in the context of general global cooling during this whole time interval^22,25,33^.

The main goals of this study are: (1) to identify the rodent metacommunities from the late Miocene of the Iberoccitanian Region and (2) analyse their spatio-temporal dynamics in relation to environmental changes as well as shifts in different ecological parameters of these metacommunities, particularly taxonomical diversity and ecological structure. In order to fulfill the first objective we used the faunal components (FC) defined by Gómez Cano, *et al*.^7^, which group genera with similar ecological affinities (see Supplementary Fig. 2). Because of the relatively homogenous ecology of their species (mainly habitat preferences), these faunal components work as environmental proxies to interpret community ecological structure. This macroecological fossil-based approach enables us to identify temporal and spatial variations of community structure, which presumably were related to changes in global and regional climate. Therefore, we used the variations in the percentages of each faunal component in each fossil association as variables for the definition of metacommunities by means of cluster analysis. We achieved the second objective through the analysis of patterns of community assembly-disassembly (as measured by nestedness) as well as the changes in taxonomic diversity and the ecological structure defined by faunal components. Subsequently, we evaluated the statistical relationship between these patterns of ecological change in metacommunities and climatic changes (as independently measured by the global isotopic record, which is related to variations in temperature and, secondarily, in precipitation patterns). Finally, since there are significant environmental differences between the southern and northern provinces within the Iberoccitanian Region^16,34,35^, we analysed community changes separately for each biogeographic province.

## Results

### Metacommunities analyses

**Figure 1:**
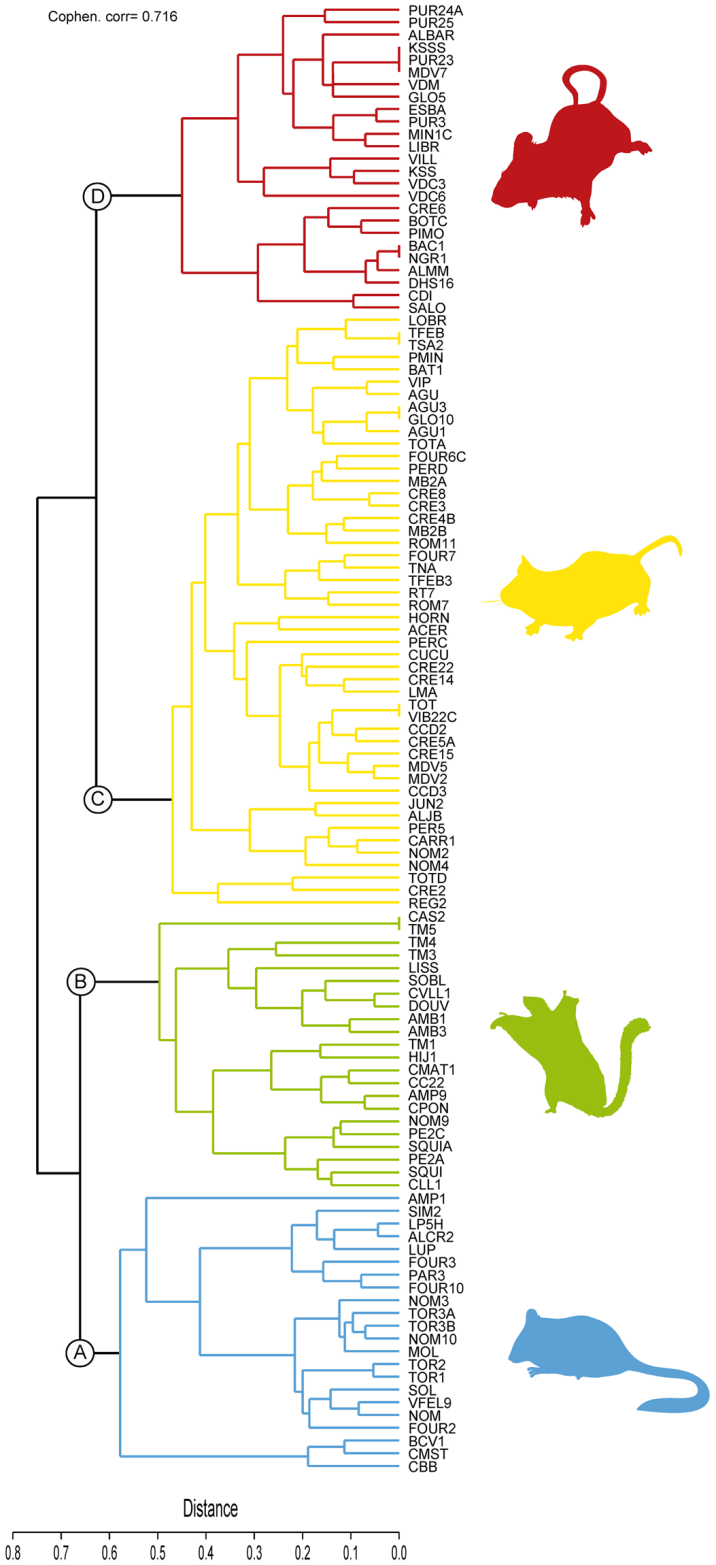
Rodent metacommunities cluster for the Iberoccitanian region during the latest middle Miocene to the Mio-Pliocene boundary. Metacommunities A, B, C and D are represented with different colours. Euclidean distance between nodes is shown at the bottom of the cluster and the coefficient of cophenetic correlation is shown at the top. Abbreviations for fossil sites as in Supplementary Table 1.

**Figure 2:**
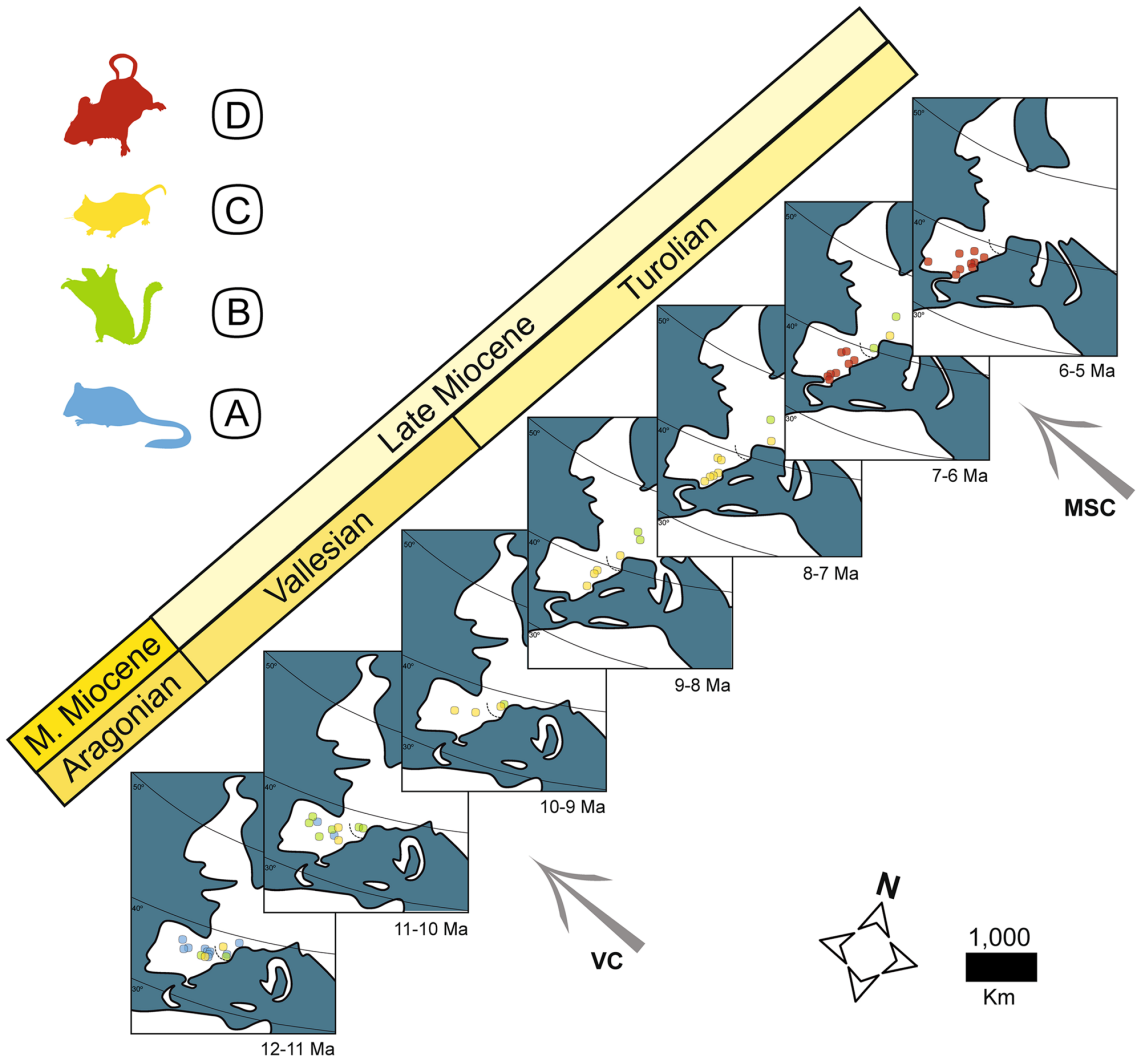
Geographical and temporal replacement of rodent metacommunities in the Iberoccitanian Region from the latest middle Miocene to the Mio-Pliocene boundary. Each map represents one million of years, except the ones in the extremes of the temporal gradient. The colours of the different fossil sites correspond to the colours assigned to each metacommunity in Fig. 1. The arrows correspond the Vallesian Crisis (VC) and Messinian Salinity Crisis (MSC). Dotted line separate the northern and southern province. Figure created with Adobe Illustrator CS6 version 16.0.0.

The cluster analysis identified four significant metacommunities (Fig. 1), which were identified with capital letters from A to D. Each metacommunity presented changes in the relevance of the different faunal components within its rodent associations (see Supplementary Table 2). Metacommunity A included fossil sites in which FC VI and FC V were dominant, sometimes accompanied by FC III. Sites included in metacommunity B were clearly dominated by taxa included in FC II. Metacommunity C was mainly dominated by FC III, with some relevance of FC II in the northern province. Finally, sites included in metacommunity D were restricted to the southern province in our database, and included higher percentages of species from the FC I and IV. The spatiotemporal dynamics of these rodent assemblages (Fig. 2) showed an initial dominance of metacommunity A in the Iberoccitanian Region with metacommunities B and C less represented. Progressively, during the Vallesian (around 11 to 9 Ma), the A metacommunity lost importance in favour of B and C in an incremental way. During the early Turolian (around 8.5 Ma), the metacommunity C was dominant in the Iberian Peninsula, and there was a northward shift of metacommunity B. Finally, the appearance of metacommunity D during the late Turolian (around 7.0 Ma) is associated to the north displacement of metacommunities B and C (Fig. 2). Geographical shifts or disappearance of these metacommunities in the northern province during the latest Turolian could not be assessed due to lack of record in this area between 6 and 5 Ma.

The nestedness analyses indicated a statistically significant nested pattern in the rodent assemblages from both bioprovinces, as well as in most of their different faunal components (Table 1).

**Table 1:** Nestedness analyses for complete Iberoccitanian late Miocene rodent assemblages (Total). and for each faunal component (FC) in southern (left) and northern (right) provinces.

	***T***	***RT***	***p***		***T***	***RT***	***p***
South	20.456	31.756	<0.001	North	22.990	37.946	<0.001
FC I	1.915	20.85	<0.001	FC I	—	—	—
FC II	17.335	30.255	0.004	FC II	12.569	28.062	<0.001
FC III	32.298	43.200	0.005	FC III	15.449	34.199	0.001
FC IV	7.054	26.157	<0.001	FC IV	—	—	—
FC V	6.243	25.558	<0.001	FC V	6.022	28.544	<0.001
FC VI	9.999	34.77	<0.001	FC VI	7.535	25.931	0.003

*T*, matrix temperature; RT, Random *T*, mean matrix temperature for 10000 randomly shuffled matrices; *p*, probability values based on the comparison between *T* and its distribution for 10000 randomly shuffled matrices.

### Diversity dynamics

**Figure 3:**
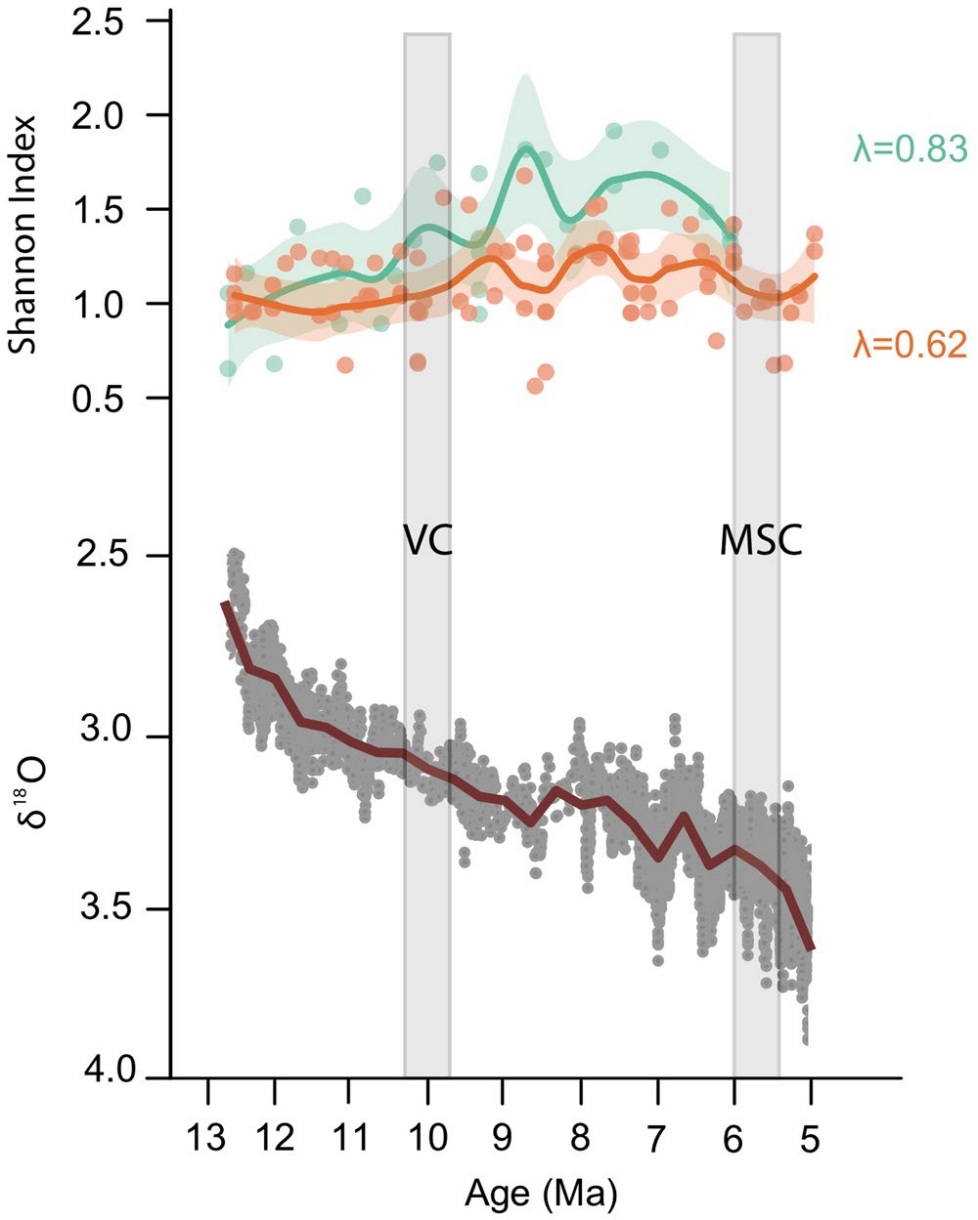
Changes in taxonomical diversity (above). Measured by Shannon index, for Iberoccitanian late Miocene rodent assemblages from the northern (blue, 27 fossil sites) and southern (orange, 90 fossil sites) provinces, compared with benthic foraminifera δ^18^O values (below) from Zachos *et al*.^33^. To visualize trends throughout the late Miocene, we applied a local regression fitting (LOESS) to diversity indexes. The smoothing parameter (λ) controls the balance between the goodness of fit of the model. Shaded areas represent the 95% confidence interval of the LOESS fit. The grey boxes represent the Vallesian Crisis (VC) and Messinian Salinity Crisis (MSC).

The Shannon diversity index increased progressively in both provinces, although this increase was slightly more pronounced in the northern province (Fig. 3). Overall, this index exhibits fluctuations through time in both provinces (Fig. 3), but in an opposite pattern, with rough synchronicity between peaks in the northern province and valleys in the southern one.

**Figure 4:**
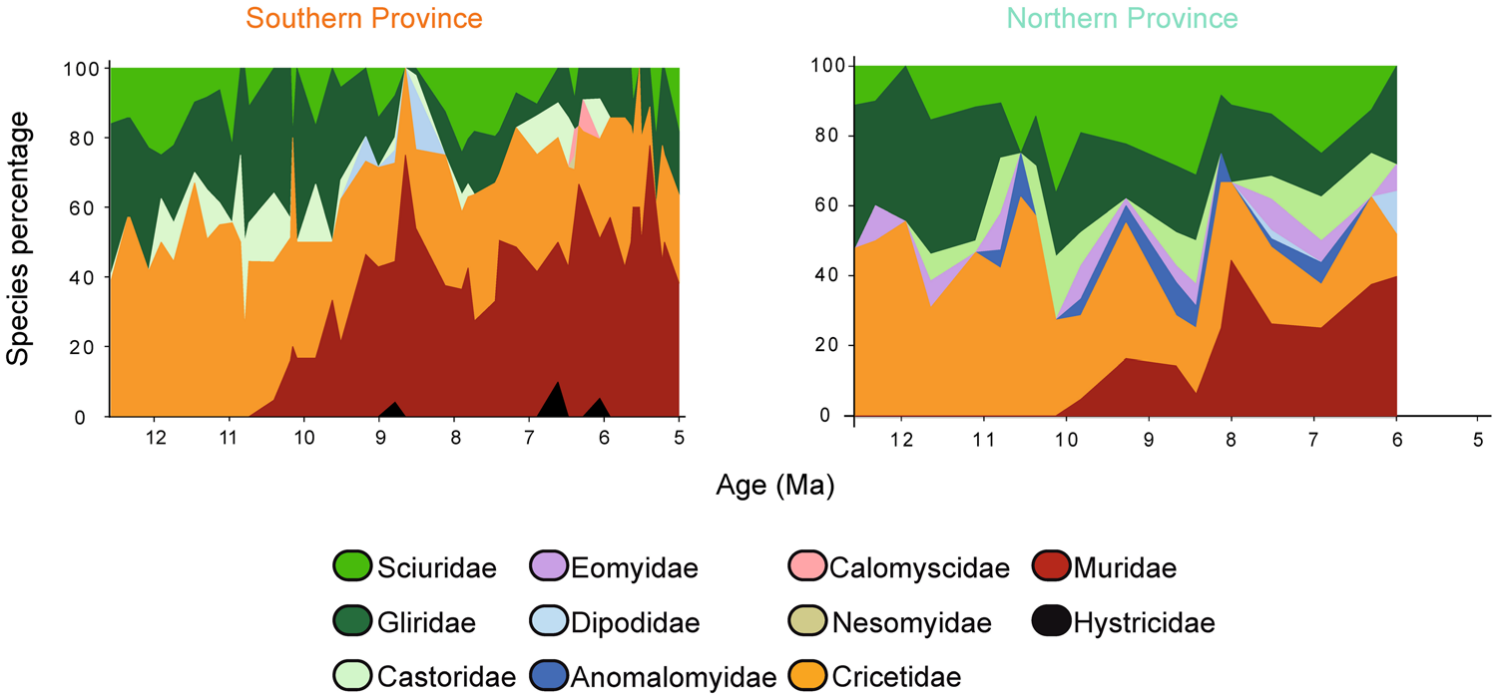
Changes in the proportions of rodent families in the Iberoccitanian Region (southern province, left; northern province, right) across the latest middle Miocene to the Mio-Pliocene boundary.

According to the dynamic of rodent families, there were similar trends in both provinces, with early dominance of Cricetidae, Sciuridae and Gliridae (Fig. 4). Around 11–10 Ma the family Muridae is recorded in the Iberoccitanian region for the first time, and became the dominant family around 9 Ma in the southern province and 1 Ma later in the northern one (Fig. 4). The other families had much lower representation in the Iberoccitanian faunas, except for the families Castoridae, and Eomyidae in the northern province (Fig. 4). It is important to note the presence of the family Hystricidae only in the southern province, where it is recorded sporadically. Nestedness ranking of rodent assemblages was significantly correlated with taxonomical diversity only in the northern province (Table 2), which indicates an ordered pattern for variations of diversity in this province and suggests that the apparent loss/gain of genera in the northern province was diversity-dependent. The negative correlation between taxonomic diversity and nestedness ranking indicates that permanence of genera in face of environmental change within this province was higher if resources were shared among more equitably distributed genera (with similar proportions of species) rather than in communities with a higher incidence of closer relatives in one or few genera. Lower variation range in the diversity index of southern fossil sites precluded a statistical relationship between diversity and nestedness ranking.

**Table 2:** Correlation between the taxonomical diversity, as measured by Shannon (H’) index with the isotopic value and nestedness ranking of the Iberoccitanian late Miocene rodent assemblages from the southern (S) and northern (N) provinces.

^δ18^O						
		σ	*p*	σ	*p*	n
H'	N	0,467	0,014	−0,610	<0,001	27
	S	0,167	0,115	−0,044	0,681	90

σ Spearman correlation coefficient.

### Environmental variables correlation

Our correlation analyses between climatic change (as measured by δ^18^O values) and the different variables studied here showed a high level of connection between environmental change and faunal dynamics.

The Shannon diversity index was not correlated with δ^18^O in the southern province, although there was a significant correlation in the northern province, which shows that the general global cooling across the late Miocene appears to be related to increases in taxonomical diversity of its rodent faunas (Table 2).

Proportion of species for most faunal components (FC) showed statistically significant correlations with isotopic values, which were slightly different in the southern and the northern province (Table 3). Proportions of FC V and VI, both related to a set of middle Miocene genera (Supplementary Fig. 2), significantly dropped in association with temperature increases in both provinces. FC III and FC IV, dominated by generalist species (Supplementary Fig. 2), correlated only marginally with isotopic variation in the southern province, while the former was significantly related to decreases in temperature in the northern province and the latter was recorded in only two northern fossil sites and statistical correlation was not possible. Finally, increases in the proportion of species of FC I, dominated by generalists and some species adapted to more open spaces (Supplementary Fig. 2), were significantly related to increases in temperature in the southern province, but this was not the case in the northern province, probably because its absence in most of the northern rodent associations precluded a robust analysis.

**Table 3:** Correlation between proportion of species richness (%S), nestedness ranking and the isotopic value of the Iberoccitanian late Miocene rodent assemblages from the southern (left) and northern (right) provinces, for all the species (total) and for those included in each faunal component (FC).

^δ18^O South					^δ18^O North				
		σ	*ρ*	n			σ	*ρ*	n
NEST	Total	0,258	0,014	90	NEST	Total	−0,188	0,347	27
	FC I	−0,196	0,182	48		FC I	—	—	—
	FC II	0,179	0,230	47		FC II	−0,016	0,942	22
	FC III	0,327	0,002	85		FC III	−0,491	0,015	24
	FC IV	−0,384	0,013	41		FC IV	—	—	—
	FC V	0,488	0,001	40		FC V	0,387	0,068	23
	FC VI	0,533	<0,001	39		FC VI	0,460	0,098	14
%S	FC I	0,291	0,045	48	%S	FC I	−0,800	0,200	4
	FC II	−0,074	0,621	47		FC II	0,300	0,165	23
	FC III	0,188	0,084	85		FC III	0,488	0,015	24
	FC IV	0,297	0,059	41		FC IV	—	—	2
	FC V	−0,490	0,001	40		FC V	−0,420	0,036	23
	FC VI	−0,817	<0,001	39		FC VI	−0,610	0,021	14

σ Spearman correlation coefficient. NEST, Nestedness correlation for total (S and N) and for theFCs

The nestedness ranking was significantly correlated with temperature in the southern province and non-significant in the northern province (Table 3). Regarding the independent FCs, in the southern province the FCs III, IV, V and VI were significantly correlated with the δ^18^O value, while in the northern province only the FC III was significantly correlated and correlations with FC V and FC VI were marginally significant (Table 3). Looking into the sign of these correlations, the decrease of temperatures provoked gains of FC IV genera in the associations from the southern province, and gain of FC III genera in the northern province. In turn, the decrease of temperatures provoked a decrease in the fossil associations of genera included in the FCs III, V and VI in the southern province, and FC V and VI in the northern province (Table 3).

## Discussion

Late Miocene rodent faunas from the Iberoccitanian region sharing similar ecological structures were classified into metacommunities, which followed non-random temporal and environmental assembly and disassembly patterns directly or indirectly related to environmental changes. Influence of climatic changes on the rodent communities proceeded through the integration of separate impacts on the different faunal components that integrated these assemblages. The proportions of species from each faunal component were significantly influenced by global temperature changes (Table 3). Additionally, our results strongly suggest that faunas from the northern and southern provinces showed differential responses to climatic changes; while temperature changes had a direct influence on the assembly-disassembly patterns of southern rodent faunas (Table 3), the influence of environmental changes appeared to be diversity-dependent in the northern province (Table 2); taxonomic diversity of rodent faunas in the northern province was influenced by temperature changes, while this was not the case for the southern province, where changes in humidity-aridity conditions might be more important^13,25^. This would be related to the effects of changes in temperature in the higher latitudes of the Iberoccitanian Region, where forested and more stable environments were dominant^7,36,37^. The temperate evergreen forests typical of the northern province in this period seem to be more affected by changes in temperature than in relative humidity^6,38^ and, therefore, the rodent faunas adapted to these environments would be indirectly affected by landscape changes induced by temperature changes. Additionally, the increase in thermal seasonality associated with latitude increase made variations in temperature more relevant than relative humidity under these Miocene tropical conditions^6,39^, since it gave rise to the lack of specific food resources (e.g. fruits) during long periods of the year. The influence of diversity-dependent dynamics on the assembly-disassembly processes of rodent metacommunities in the northern province suggests that permanence of taxa in face of environmental change within this province was lower among close relatives than if they belonged to different genera. Although the functional role of diversity has been the subject of a long-standing debate in ecology, diversity tends to be correlated positively with ecosystem stability^40–42^, which is dependent on the differential response of species or functional groups to variable conditions, as well as the functional redundancy of species that have important stabilizing roles.

On the contrary, our results on the southern province suggest that, while the diversity of the local faunas was not correlated to changes in temperature (Table 2), the structure of the communities may have been influenced mostly by temperature changes (Table 3). The significant correlations of the nestedness ranking with isotopic values showed that global temperature changes gave rise to an ordered substitution of the metacommunities and most of their components. However, some parts of that structure were possibly also determined by aridity changes. For example, there was no significant correlation between temperature changes and proportion of species in FC II (Table 3), which was dominated by forest adapted species (Supplementary Table 2). Due to the mostly dry climates of the southern province^19^, slight fluctuations of relative humidity had a crucial influence in forest environments and their associated fauna^9^.

**Figure 5:**
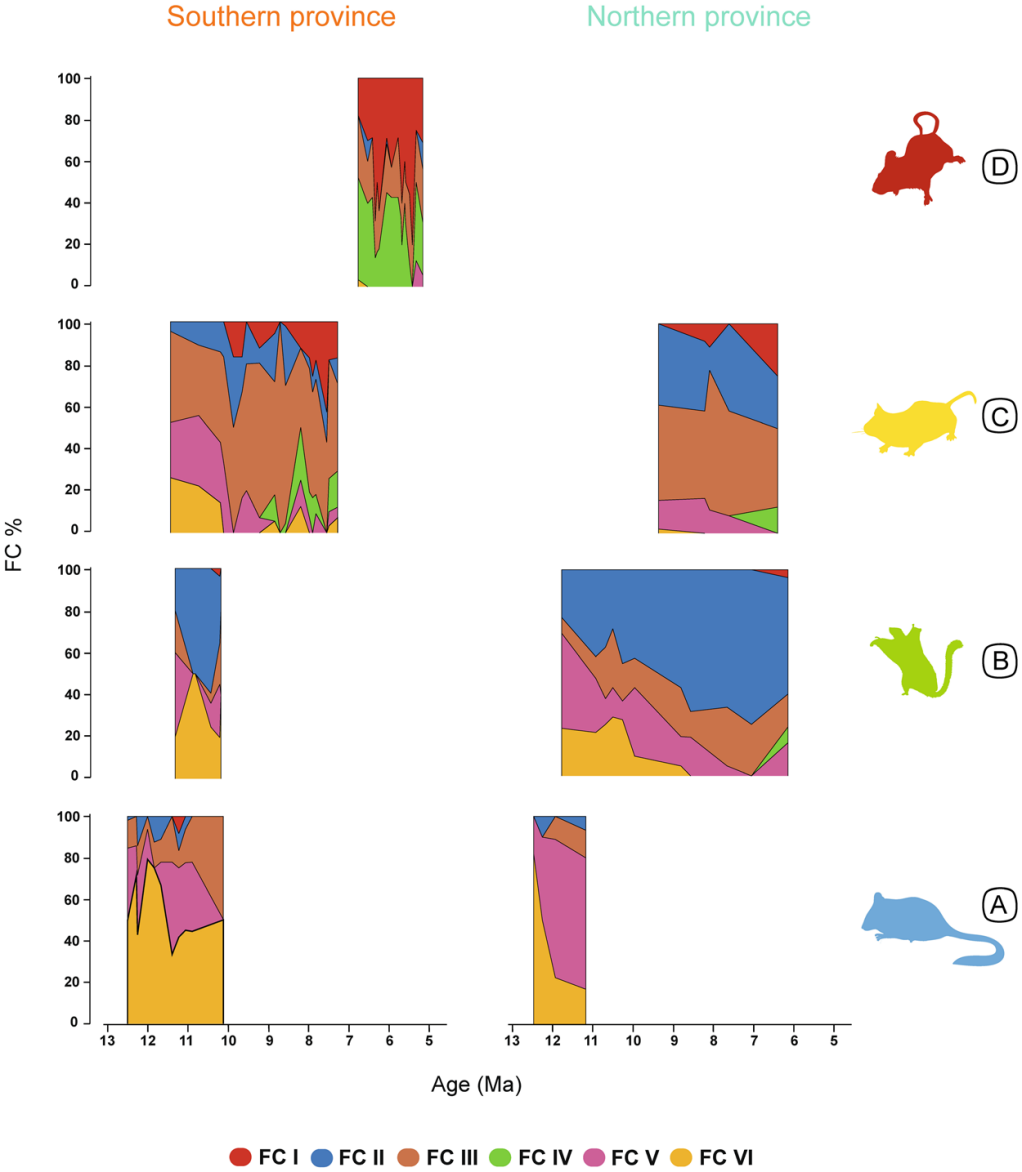
Changes in the ecological structure of each metacommunity (A–D). According to the time series of faunal components percentage values (%FC), which represent percentages of species in each component (for colours representing each FC, see the legend at the bottom of the figure). Values for southern fossil sites (right) and northern fossil sites (left) are shown separately. See supplementary material for an in-deep explanation of this figure.

In addition to the different ecological processes implied in the faunal dynamics of the provinces, faunal changes in relation to major environmental crises during the late Miocene appear to be delayed in the northern province when compared to the southern one (Figs 3 and 5). Eventually, changes in the ecological structure of rodent communities and turnover among metacommunities seem to be much more pronounced in the southern province than in the northern province (Fig. 5).

The simultaneous disappearance of metacommunities A and B and final dominance of metacommunity C in the southern province coincided with what has been called the Vallesian Crisis^43,44^, while there was a gradual substitution of metacommunities in the northern province during a much longer time interval during the late Miocene and started one million years earlier. Particularly interesting is the gradual change produced in the ecological structure of metacommunity B in the northern province across all the fossil sites studied (Fig. 5). Casanovas-Vilar and Agusti^37^ suggested that seasonality increase in the southern province was related to an exacerbated arid season while in the northern province there was simultaneous cooling and increase in aridity. These changes coincided with the gradual increase of thermal and hydric seasonality derived from the global cooling initiated in the middle Miocene^7,20,22,25,27,32^, which affected a set of species related to forested and humid environments during the Vallesian Crisis and favoured the murid immigrants. These changes in seasonality and the general trend of increasing aridity since the latest Miocene to the Pliocene gave rise to the expansion of relatively open ecosystems in the Iberian Peninsula^25,38^, which would affect Iberoccitanian rodent metacommunities by driving a substitution of the faunas dominated by dormice (metacommunity A), for a more diversified set of ubiquitous taxa, particularly within Muridae^7,45^. Additionally, the prevalence of metacommunity B in the southern province was probably related to humid pulses during the latest Aragonian (12–11 Ma) and early Vallesian (11–10 Ma)^9,18^, which allowed for the dispersal of forest elements from Central Europe through the northern province. The pulse of increment in taxonomical diversity around 10 Ma (Fig. 4) in the northern province is congruent with such a dispersal scenario and could be related to the first occurrence documented for murid taxa in this province^13^ as well as the decrease in Gliridae (Fig. 4).

While the initial concept of the Vallesian Crisis considered it as an abrupt event^44^ and relevant changes have been observed in both macro- and micromammal faunas^7,20,32^, during the last years several authors have suggested that it could really be a succession of extinctions during a more prolonged time interval^7,24,27,32^. The coexistence of three different rodent metacommunities during the early Vallesian in the southern province as well as the prolonged persistence and gradual changes in the ecological structure of metacommunity B in the northern province appear to reinforce this interpretation of the Vallesian Crisis as a gradual event. Additionally, according to our results, the Vallesian Crisis rendered different outcomes in the southern and northern provinces of the Iberoccitanian Region, with the disappearance of metacommunities A and B in the former and the appearance of metacommunity C in the latter (Figs 2 and 5).

A second peak of diversity around 8.5 Ma in the northern province (Fig. 3) could be related to the displacement of metacommunity B towards higher latitudes within the northern province (Fig. 2), while metacommunity C became dominant in the Iberian Peninsula. The latter was mostly composed by murids, which were involved in a sudden diversification in Europe during the late Miocene^37,46^. This time was another moment of global cooling and aridification^13^, which favoured the survival and diversification of murids, probably due to their generalist condition. Nevertheless, regarding the southern province, taxonomic diversity in rodent faunas dropped around this date (Fig. 3), which was due to the relevance of rodent faunas highly dominated by numerous murid species (Fig. 4). The sudden reversion in the diversity trends in both the northern and southern provinces close to 8 Ma could be related to a recovery of previous climatic values (Fig. 4).

Noteworthy changes in rodent communities from the southern province around 7 Ma during the late Turolian were associated with the beginning of the Messinian, around one million years before the Messinian Salinity Crisis (Fig. 5). Metacommunity D appeared in the southern province (Figs 2 and 5) and completely displaced metacommunity C, which only remained in the northern province due to the arid conditions dominant in the south and is involved in the last diversity decrease observed in the northern province (Fig. 3). Unfortunately, there is no record of rodent faunas in the northern province after this time period and the development of these metacommunities in that province is unknown at this moment. The sudden dominance of metacommunity D in the southern province could be related to the progression of decreasing global temperatures and aridity increase that, through glacio-eustatic sea-level changes, is at least partially responsible for the onset of the Messinian Salinity Crisis (MSC) around 6 Ma^22^. The MSC event represented a drastic increase in aridity, which affected the general pattern of faunal turnover in the Mediterranean region^7,22^ and reduced taxonomical diversity of rodent faunas from the southern province (Fig. 3), largely dominated once more by murid species.

Our work demonstrates that the influence of global and regional climatic changes on metacommunity dynamics is not simple and depends on the environmental characteristics of the affected areas as well as the characteristics of the species present in the community. In general, we found evidence of direct and indirect environmental controls on the variations of the ecological structure of rodent metacommunities, which were not only linked to temperature changes, but there was probably also an influence of the changes in aridity that occurred in this region during the late Miocene, particularly in the southern province. However, the diversity of rodent communities was more influenced by temperature than by humidity-aridity conditions in the northern province, through the effects of temperature and thermal seasonality changes on the dominant forest environment. Due to its buffer environmental conditions, the northern province of the Iberoccitanian region, or at least parts of it, played an important role as a humid fauna refuge where the metacommunities B and C survived successive aridity increases during the late Vallesian and the Turolian, before their alleged disappearance, which also evidences the stabilization effects of the higher diversity of the northern rodent faunas.

Although they were not drastic, we also found evidence of changes in the ecological structure of rodent faunas linked to the Vallesian and Messinian crises, particularly concerning the taxonomical diversity of the assemblages. We observed that diversity values were less variable in the southern province during the late Miocene, which is probably linked to the fact that aridity-prone faunas dominated rodent assemblages in this province. Notwithstanding, weak fluctuations in the Shannon diversity index through time in the southern sites were opposite to those in the northern province, which is consistent with the contrasting differences between these biogeographic provinces and their associated faunas.

## Methods

### Study area and fossil sites

The study area of this work spans the Iberoccitanian Region (Supplementary Fig. 1), comprising 117 fossil localities from the latest middle Miocene to the earliest Pliocene (12.6 to 4.9 Ma). This region is a key area area for the development of macroevolutionary studies due to the completeness, richness and abundance of fossil remains found in long-term and continuous stratigraphic sequences^14,47,48^. The study area exhibits strong environmental differences from the rest of Europe, which persisted in time due to its isolated position in the westernmost part of Europe^22,49^. Furthermore, there are two environmentally distinctive mammalian bioprovinces in this region^16,34^, recognizable since the Eocene^35,50^. The northern province includes fossil sites from the Rhône, Provence, Cucuron-Basse Durance and Languedoc-Rousillon basins from South-Eastern France, and the Vallés-Penedés basin from Catalonia (northeastern Spain). All the other fossil sites from the Iberian Peninsula are included in the southern province and are located at the Alfambra-Teruel, Alicante, Baixo Tejo, Castellón, Calatayud-Daroca, Duero, Fortuna, Granada, Guadix-Baza, Hijar, Murcia, Tajo, and Valencia basins (Supplementary Fig. 1).

### Data base

Our database is derived from Gómez Cano^6^ and considers all rodent species recorded at the studied fossil sites, including 209 species. In order to reduce sampling biases, this database included well sampled fossil sites, which recorded a minimum of 100 m1 + m2 + M1 + M2, following van der Meulen and Daams^18^. Only a few fossil sites with a lower sample size were allowed as they were the only representatives of poorly sampled areas or because they were part of stratigraphically important sequences.

We employed this dataset of rodent species to compile a matrix with information on the number of species and percentage of species of each genus and family in each fossil site. As in other studies^16^, we only analysed taxa that were determined at the species level in each fossil site to avoid potential noise in the data due to unidentified taxa. We also assigned all the species in our matrix into the faunal components (FC) defined by Gómez Cano, *et al*.^7^, which grouped genera with similar ecological affinities (Supplementary Fig. 2).

Finally, we also compiled independent bibliographic information for isotopic data contemporary to fossil sites included in our work^7^; this work follows Gómez Cano, *et al*.^7^ by fitting a smoothed curve to the isotopic information provided by Zachos, *et al*.^51^ and interpolate δ^18^O values for the age of each fossil site. We used the global marine isotopic record because the continental isotopic record from the Iberoccitanian Region has low resolution in comparison to the rodent fossil record, although it is increasing at a steady pace^52–55^. Global isotopic record allow us to identify minimum ecological changes that affected rodent faunas.

### Metacommunity identification

In order to identify different metacommunities in the Iberoccitanian region during the studied time interval, we carried out a cluster analysis including the data from both bioprovinces.

Since we sought to group fossil sites according to the ecological affinities of their species instead of the taxonomical resemblance between them, we used faunal components as the studied variables^7^. These faunal components group together rodent genera with relatively similar ecological characteristics (as shown in previous works, see Supplementary Fig. 2 and references therein), which showed similar responses through time during the late Miocene^7^. Therefore, we calculated the percentage of species for every faunal component registered in each fossil site in relation to the total number of species in such site. The use of species percentages instead of the number of species allows the homogenization of all fossil sites for comparisons among them despite differences in species richness, which minimizes potential sampling biases (although they cannot be completely avoided).

The cluster analysis was calculated on Euclidean distances between group centroids and the clustering procedure was UPGMA (Unweighted Pair Group Method with Arithmetic Mean, also called Paired group) using the PAST 3.11 software^56^. Thereafter, we used the NbClust package of R software^57^ to identify the significant number of groups within each cluster, which defined the number of metacommunities identified.

### Diversity dynamics

We calculated the Shannon index (H’) to assess how diversity was related to changes in environment and ecological structure of rodent faunas within different metacommunities. This index takes into account the evenness of a dataset, so the cases with equitable numbers of entities between different types have higher diversity values. In ecology of modern ecosystems communities are customarily the studied cases, the types of interest are usually species and the entities of interest are commonly measured as number of individuals^58,59^. Nevertheless, since there are no available data on number of individuals for each species for all the studied fossil sites, and multiple taphonomic biases can affect to the representativity of proxies to individual abundance (such as number of identified specimens, inferred minimum number of individuals or number of molars), we used an approach that reflects taxonomical diversity rather than ecological diversity; we used the proportion of species (abundance of entities) for each genus (types) to calculate the Shannon index (H’) for each one of the fossil sites (cases) in the two provinces. This measure gives an idea of evolutionary diversification within communities, establishing a gradient between communities in which resources are shared among a more or less equitable number of lineages. In order to study how this index changes through time we plotted the values for each one of the fossil sites against time and applied a local regression fitting (LOESS) over the data to visualize their trends throughout time. Finally, we also computed proportion of species in each faunal component as an additional biodiversity measure.

Since it is commonly proposed as a general model of community disassembly^60^, we also evaluated the possible presence of a nested structure in the assembly and disassembly patterns observed in the Iberoccitanian rodent faunas during the latest middle Miocene to Mio-Pliocene boundary. This model proposes that communities within disturbed systems exhibit nested structure such that the taxa included in poorer communities represent a confined subset of those in richer assemblages, rather than a random selection of those found in the entire species pool^61–63^. This pattern would imply that each taxon requires some minimal conditions to support population levels adequate to resist extinction, and that it can occur in all sites that attain these conditions. We calculated the nestedness of the rodent assemblages following the algorithm proposed by Rodríguez‐Gironés and Santamaría^64^ based on data of genus presence–absence matrices arranged by genus richness and number of occurrences. Following Furió, *et al*.^10^,we used genera in order to avoid the possible noise derived from the species multiplicity within the fossil sites among sedimentary basins in both provinces^10^. Additionally, since species go extinct over a few m.y., in order to conduct the research over a long span of time, we have to use higher taxonomic units than species, and it is usually considered that congeneric species were ecologically similar. Finally, rodent taxonomy may change at the species level (e.g. difficulties in species differentiation due to anagenesis may become a problem in some lineages) but it is very consistent at the genus level.This algorithm calculates the nested subset temperature (a nestedness score) of each matrix in such a way that the lower the score, the more nested the structure of the community^65^. We calculated *p* values by means of a comparison to the distribution of scores generated by randomly shuffling the original matrices through 10000 Monte Carlo simulations (rows and sum totals were maintained constant). Nestedness analyses were run using the nestedness function as implemented in the R library Bipartite^57^ and the null model 3 as suggested by Rodríguez‐Gironés and Santamaría^64^, which is a constrained null model that accounts for the frequency of genera (column totals) and the genus richness of fossil sites (row totals) while sampling the null space uniformly, which minimises type I and II errors. Finally, when significant nestedness was identified, we compared the rank order in which assemblages were nested to their order based on diversity index and isotopic value using Spearman’s rank correlation^66,67^. These analyses were performed for both bioprovinces as well as using independent matrices corresponding to the genera included in each faunal component^8^.

### Influence of climatic change

Finally, In order to evaluate the relevance of climate change in the establishment of the different metacommunities and their ecological and taxonomical characteristics (diversity and nestedness), we performed correlation analyses of the different measurements previously commented with the variations in the δ^18^O isotopic record as interpolated from Zachos, *et al*.^51^ for the age of each fossil site included in this paper.

## Electronic supplementary material

Supplementary information
