# Supplementary material for: Differential responses of Miocene rodent metacommunities to global climatic changes were mediated by environmental context
Source: Sci Rep. 2018 Feb 6;8:2502. doi: 10.1038/s41598-018-20900-5 (PMC5802738; doi:10.1038/s41598-018-20900-5)
Supplement: Supplementary file 1 — Supplementary information [file 41598_2018_20900_MOESM1_ESM.pdf]

Differential responses of Miocene rodent metacommunities to global climatic changes were mediated by environmental context

Fernando Blanco[a*], Ana R. Gómez Cano[b,c], Juan L. Cantalapiedra[d], M. Soledad Domingo[a,e], Laura Domingo[a,f,g], Iris Menéndez[a,g], Lawrence J. Flynn[h] and Manuel Hernández Fernández[a,g]

[a] Departamento de Geodinámica, Estratigrafía y Paleontología, Facultad de Ciencias Geológicas, Universidad Complutense de Madrid. C/ José Antonio Novais 12, 28040 Madrid (Spain)

[b] Transmitting Science. C/Gardenia 2, Piera, 08784, Barcelona (Spain)

[c] Institut Català de Paleontologia Miquel Crusafont. Edifici ICP, Campus de la UAB s/n, 08193 Cerdanyola del Vallès (Spain)

[d] Museum für Naturkunde, Leibniz-Institut für Evolutions und Biodiversitätsforschung. Invalidenstraße 43, 10115 Berlin (Germany)

[e] Departamento de Ecología Evolutiva, Estación Biológica de Doñana (CSIC). C/Américo Vespucio 26, 41092 Seville (Spain)

[f] Earth and Planetary Sciences Department, University of California Santa Cruz. 1156 High Street, CA 95064 (USA)

[g] Departamento de Cambio Medioambiental, Instituto de Geociencias (UCM, CSIC). C/ José Antonio Novais 12, 28040 Madrid (Spain)

[h] Department of Human Evolutionary Biology, Harvard University. 11 Divinity Avenue., Cambridge, MA 02138 (USA)

Corresponding author

Fernando Blanco

Departamento de Paleontología

Facultad de Ciencias Geológicas

Universidad Complutense de Madrid

C/ José Antonio Novais 12, 28040, Madrid (Spain)

e-mail: fernandoblanco@ucm.es

Supplementary text

We found metacommunity A as dominant at the beginning of the time interval studied (Fig. 2). It was mostly composed by taxa within FC V, in the north and mostly VI in the south (Fig. 5), both dominated by dormice (Gliridae) [1]; the northern fossil sites were dominated by forest glirids (FC V, Supplementary Fig. 2), while the southern ones included more glirid species mostly adapted to open environments (FC VI, Supplementary Fig. 2). Since FC V was the most abundant one in the northern province during latest middle Miocene, there is a concordance with the presence of forest environments [2]. Codominance of FC V and VI in the southern province suggests an increase of environmental humidity, and coincides with the development of the metacommunity B. We also found FC III, which is defined by generalist faunas adapted to both open and forest environments (Supplementary Fig. 2), associated to metacommunity A in relatively high proportions towards the end on this metacommunity in the southern province, which would announce the subsequent metacommunity C. Our results showed a progressive disappearance of A, which spanned the late Aragonian to the end of the early Vallesian (Fig. 2). This metacommunity was replaced by more diversified associations, which could suggest a decline of dormice due to competition with other rodents [3-7]. However, some generalist glirids (e.g. *Eliomys*) survived after 11 Ma (Fig. 2) and were able to overcome the increase in aridity during the late Miocene [8], which suggests a relevant role for environmental forcing.

At the beginning of the Vallesian there was a significant replacement when the metacommunity B turned dominant, especially in the north (Fig. 2 and 5). FC II, which is defined by its highly diverse association of forest adapted taxa (such as *Ramys* or *Blackia*, Supplementary Fig. 2), was dominant in the northerly associations of the metacommunity B. The relatively few fossil sites in the southern province that were included in this metacommunity showed substantial proportions of FC VI, as a reminiscence of metacommunity A. The short temporal relevance of this metacommunity in the southern province suggests that it might be considered as a transitional phase between metacommunities A and C.

Before the disappearance of metacommunities A and B in the southern province we found during the latest Aragonian the appearance of metacommunity C, which mainly comprised the ubiquitous faunas of FC III, such as *Hispanomys*, *Spermophilinus* or

Tempestia, adapted to open woodland areas [1]. This metacommunity became dominant around 10 Ma, after the decline of the previous metacommunities dominated by faunas adapted to humid and forest conditions (metacommunities A and B). Towards the end of its duration this metacommunity included a substantial proportion of taxa included in FC I, some of which showed preference for arid environments, such as the terrestrial squirrel *Atlantoxerus*, or with a generalist character such as the dormouse *Eliomys* and the mice *Stephanomys* or *Castillomys* [9,10], which indicates the transitional character of these final associations of metacommunity C (Supplementary Fig. 2). Noteworthy, in the northern province we also found some forest adapted faunas composed by FC II taxa such as *Miopetaurista*, *Eomyops* or *Rotundomys* [10].

Finally, metacommunity D appeared in the southern province during the late Turolian (Fig. 3 and 5), and became dominant after that. It comprised taxa mostly included in FCs I and IV, which grouped both arid and ubiquitous faunas (Supplementary Fig. 2), such as *Atlantoxerus*, *Debruijnimys*, *Myocricetodon*, *Castromys*, *Castillomys*, *Apocricetus*, or *Ruscinomys* [1].

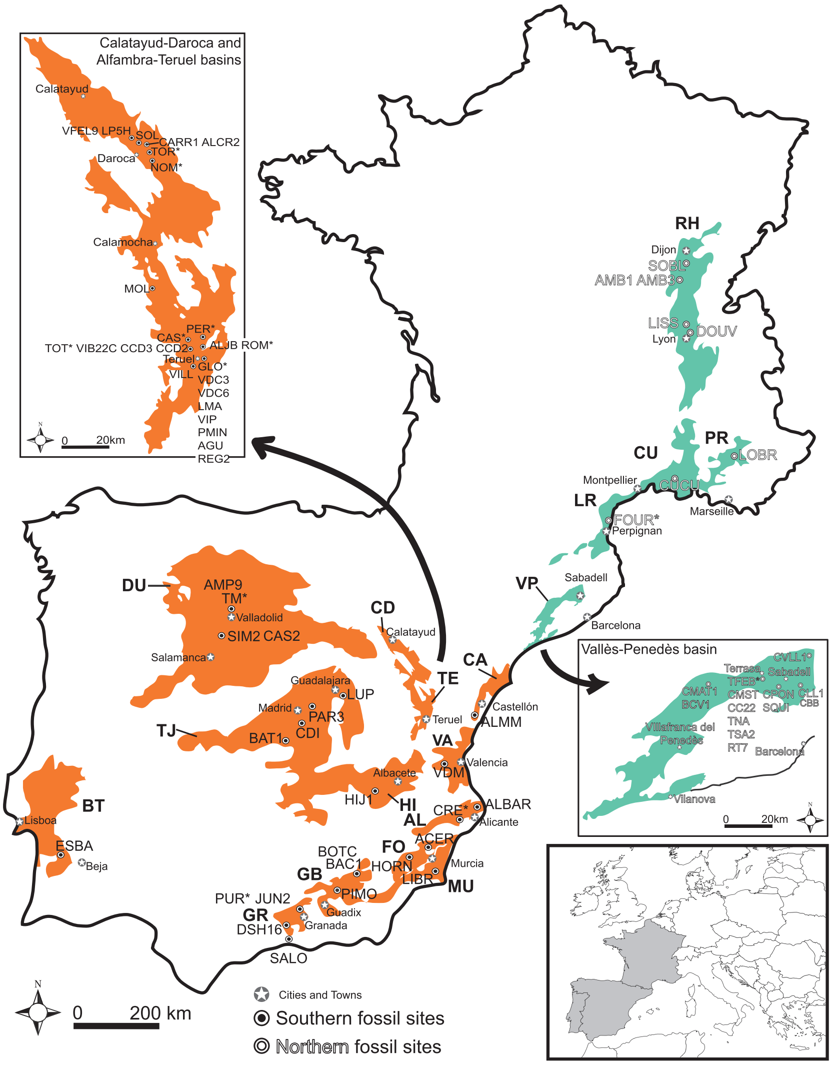

Supplementary Figure 1. Iberoccitanian fossil sites from the latest middle Miocene to the Mio-Pliocene boundary considered in this study. Blue basins, northern biogeographic province; Orange basins, southern biogeographic province; *, stratigraphic sections with several sites; see Supplementary Table 1 for abbreviations for basins (bold) and fossil sites (regular). Modified with Adobe Illustrator CS6 version 16.0.0 from Gómez Cano [11].

Ecological significance

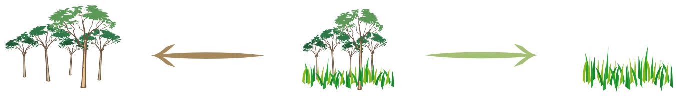

Faunal component	Forest specialists		Generalists		Open landscapes specialists	Metacommunities<br>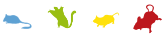<br>A	B	C	D
I	*Micromys*<br>*Paraethomys*		*Eliomys*<br>*Calomyscus*<br>*Dendromus*<br>*Trilophomys*<br>*Myocricetodon*<br>*Castillomys*<br>*Stephanomys*	*Atlantoxerus*	*Debruijnimys*<br>*Pseudomeriones*				✔
II	*Albanensia*<br>*Blackia*<br>*Hylopetes*<br>*Miopetaurista*<br>*Pliopetaurista*<br>*Glirulus*<br>*Glis*<br>*Eomyops*<br>*Keramidomys*<br>*Eozapus*<br>*Neocricetodon*	*Chalicomys*<br>*Palaeomys*<br>*Trogontherium*	*Tamias*<br>*Graphiurops*<br>*Ramys*<br>*Microtocricetus*<br>*Cricetulodon*<br>*Rotundomys*<br>*Anomalomys*<br>*Prospalax*	*Epimeriones*			✔	✔	
III	*Huerzelerimys*	*Hispanomys*	*Spermophilinus*<br>*Tempestia*<br>*Apodemus*<br>*Occitanomys*<br>*Progonomys*					✔	
IV		*Dipoides*<br>*Blancomys*	*Ruscinomys*<br>*Apocricetus*<br>*Anthracomys*<br>*Castromys*<br>*Rhagapodemus*<br>*Hystrix*						✔
V	*Bransatoglis*<br><br>*Glirudinus*<br>*Myoglis*<br>*Eomuscardinus*<br>*Muscardinus*<br>*Paraglirulus*<br>*Eumyarion*	*Democricetodo*<br>*n*	*Myomimus*			✔			
VI	*Megacricetodon*		*Palaeosciurus*<br>*Cricetodon*	*Heteroxerus*<br>*Armantomys*<br>*Microdyromys*<br>*Miodyromys*<br>*Pseudodryomys*		✔			

101 Supplementary Figure 2. Taxonomical composition of rodent faunal components from the Iberoccitanian late Miocene [1]. Ecological characterization of genera can be found in Casanovas-Vilar and Agusti [2], García-Alix, et al. [9], Gómez Cano, et al. [12], Colombero, et al. [13], Daams and de Bruijn [14], Daams and Van der Meulen [15], Daams, et al. [16], van Dam and Weltje [17], de Bruijn and Ünay [18], de Bruijn [19], Fejfar [20], Freudenthal and Mein [21], Hernández Fernández and Peláez-Campomanes [22], Kälin [23], Martín-Suárez, et al. [24], Mein [25], Rummel [26] and Wessels [27]. Ticks indicate major relevance of a faunal component in the late Miocene rodent metacommunities from southwestern Europe, as shown in Supplementary table 2. Trees and grass drawings were created by Adobe Illustrator CS6 version 16.0.0.

Supplementary Table 1. Fossil sites included in the analyses for the Iberoccitanian region. Basin abbreviations: TE. Alfambra-Teruel; AL. Alicante; BT. Baixo Tejo; CA. Castellón; CD. Calatayud-Daroca; CU. Cucuron-Basse Durance; DU. Duero; FO. Fortuna; GR. Granada; GB. Guadix-Baza; HI. Hijar; LR. Languedoc-Roussillon; MU. Murcia; PR. Provence; RH. Rhône; TJ. Tajo; VA. Valencia; VP. Vallès Penedès.

**Basin**	**Fossil site**	**Fossil site abbreviation**
FO	Casa del Acero	ACER
TE	Aguanaces	AGU
TE	Aguanaces 1	AGU1
TE	Aguanaces 3	AGU3
AL	Alcoy Barranco	ALBAR
CD	Alcocer 2	ALCR2
TE	Aljezar B	ALJB
CA	Almenara M	ALMM
RH	Amberieu 1	AMB1
RH	Amberieu 3	AMB3
DU	Ampudia 1	AMP1
DU	Ampudia 9	AMP9
GB	Bacochas 1	BAC1
TJ	Batallones 1	BAT1
VP	Barranc de Can Vila 1	BCV1
GB	Botardo C	BOTC
CD	Carrilanga 1	CARR1
DU	Casasola 2	CAS2
VP	Castell de Barberà	CBB
VP	Creu Conill 22	CC22
TE	Concud 2	CCD2
TE	Concud 3	CCD3
TJ	Canteras de Iberia	CDI

VP	Can Llobateres 1	CLL1
VP	Can Mata I (Bretxa de Can Mata)	CMAT1
VP	Can Misert	CMST
VP	Can Ponsic	CPON
AL	Crevillente 14	CRE14
AL	Crevillente 15	CRE15
AL	Crevillente 17	CRE17
AL	Crevillente 2	CRE2
AL	Crevillente 22	CRE22
AL	Crevillente 3	CRE3
AL	Crevillente 4B	CRE4B
AL	Crevillente 5A	CRE5A
AL	Crevillente 6	CRE6
AL	Crevillente 8	CRE8
CU	Cucuron	CUCU
VP	Can Vilella 1	CVLL1
GR	Dehesa 16	DHS16
RH	Douvre	DOUV
BT	Esbarrondoiro	ESBA
LR	Lo Fournas 10	FOUR10
LR	Lo Fournas 2	FOUR2
LR	Lo Fournas 3	FOUR3
LR	Lo Fournas 6C	FOUR6C
LR	Lo Fournas 7	FOUR7
BT	Freiria do Rio Maior	FRM
DU	Los Valles de Fuentidueña	FUDU
TE	La Gloria 10	GLO10
TE	La Gloria 5	GLO5
HI	Hijar 1	HIJ1
FO	Hornera	HORN

GR	Jun2	JUN2
TE	Las Casiones	KSS
TE	Las Casiones superior	KSSS
MU	Librilla	LIBR
RH	Lissieu	LISS
TE	Los Mansuetos	LMA
PR	Lobieu	LOBR
CD	Las Planas 5H	LP5H
TJ	Lupiana	LUP
TE	Masia del Barbo 2A	MB2A
TE	Masia del Barbo 2B	MB2B
TE	Masada del Valle 2	MDV2
TE	Masada del Valle 5	MDV5
TE	Masada Del Valle 7	MDV7
VA	Los Mingos 1C	MIN1C
TE	Molina de Aragón	MOL
GB	Negratín 1	NGR1
CD	Nombrevilla (classical)	NOM
CD	Nombrevilla 10	NOM10
CD	Nombrevilla 2	NOM2
CD	Nombrevilla 3	NOM3
CD	Nombrevilla 4	NOM4
CD	Nombrevilla 9	NOM9
TJ	Paracuellos 3	PAR3
CD	Peralejos 2A	PE2A
CD	Peralejos 2C	PE2C
TE	Peralejos 5	PER5
TE	Peralejos C	PERC
TE	Peralejos D	PERD
GB	Pino Mojón	PIMO

TE	Puente Minero	PMIN
GR	Purcal 23	PUR23
GR	Purcal 24A	PUR24A
GR	Purcal 25	PUR25
GR	Purcal 3	PUR3
TE	Regajo 2	REG2
TE	Masia la Roma 11	ROM11
TE	Masia la Roma 7	ROM7
VP	Autopista Rubí-Terrasa 7	RT7
GR	Salobreña	SALO
DU	Simancas 2	SIM2
RH	Soblay	SOBL
CD	Solera	SOL
VP	Sant Quirze	SQUI
VP	Sant Quirze A	SQUIA
VP	Torrent de Febulines	TFEB
VP	Torrent de Febulines 3	TFEB3
DU	Torremormojón 1	TM1
DU	Torremormojón 3	TM3
DU	Torremormojón 4	TM4
DU	Torremormojón 5	TM5
VP	Trinxera Nord Autopista	TNA
CD	Toril 1	TOR1
CD	Toril 2	TOR2
CD	Toril 3A	TOR3A
CD	Toril 3B	TOR3B
TE	Tortajada	TOT
TE	Tortajada A	TOTA
TE	Tortajada D	TOTD
VP	Trinxera Sud Autopista II	TSA2

TE	Valdecebro 3	VDC3
TE	Valdecebro 6	VDC6
VA	Venta del Moro	VDM
CD	Villafeliche 9	VFEL9
TE	Villalba baja 22C	VIB22C
TE	Villastar	VILL
TE	Vivero de Pinos	VIP

Supplementary Table 2. Species percentages for Faunal Components (%FC) in each fossil site. Fossil site abbreviation correspond to Supplementary Table 1. Metacommunity letters as represented in Figure 1.

Northern Fossil site	Metacommunity	%FCI	%FCII	%FCIII	%FCIV	%FCV	%FCVI
AMB1	B	0.000	0.667	0.286	0.000	0.048	0.000
AMB3	B	0.000	0.750	0.250	0.000	0.000	0.000
BCV1	A	0.000	0.000	0.111	0.000	0.667	0.222
CBB	A	0.000	0.133	0.067	0.000	0.667	0.133
CC22	B	0.000	0.286	0.286	0.000	0.143	0.286
CLL1	B	0.000	0.429	0.143	0.000	0.333	0.095
CMAT1	B	0.000	0.375	0.250	0.000	0.125	0.250
CMST	A	0.000	0.000	0.200	0.000	0.600	0.200
CPON	B	0.000	0.455	0.182	0.000	0.091	0.273
CUCU	C	0.250	0.250	0.375	0.125	0.000	0.000
CVLL1	B	0.000	0.667	0.167	0.000	0.167	0.000
DOUV	B	0.000	0.688	0.125	0.000	0.188	0.000
FOUR10	A	0.000	0.000	0.000	0.000	0.111	0.889
FOUR2	A	0.000	0.100	0.000	0.000	0.400	0.500
FOUR3	A	0.000	0.000	0.000	0.000	0.250	0.750

FOUR6C	C	0.111	0.111	0.667	0.000	0.111	0.000
FOUR7	C	0.083	0.333	0.417	0.000	0.167	0.000
LISS	B	0.077	0.462	0.154	0.154	0.154	0.000
LOBR	C	0.000	0.417	0.500	0.000	0.083	0.000
RT7	C	0.000	0.286	0.429	0.000	0.286	0.000
SOBL	B	0.000	0.571	0.238	0.000	0.143	0.048
SQUI	B	0.000	0.421	0.105	0.000	0.263	0.211
SQUIA	B	0.000	0.231	0.077	0.000	0.462	0.231
TFEB	C	0.000	0.429	0.571	0.000	0.000	0.000
TFEB3	C	0.000	0.400	0.300	0.000	0.200	0.100
TNA	C	0.000	0.400	0.400	0.000	0.200	0.000
TSA2	C	0.000	0.429	0.571	0.000	0.000	0.000

Southern Fossil site	Metacommunity	%FCI	%FCII	%FCIII	%FCIV	%FCV	%FCVI
ACER	C	0.429	0.143	0.429	0.000	0.000	0.000
AGU	C	0.000	0.286	0.571	0.143	0.000	0.000
AGU1	C	0.000	0.333	0.667	0.000	0.000	0.000
AGU3	C	0.000	0.286	0.714	0.000	0.000	0.000
ALBAR	D	0.250	0.000	0.250	0.375	0.125	0.000
ALCR2	A	0.000	0.000	0.125	0.000	0.125	0.750
ALJB	C	0.125	0.000	0.375	0.250	0.125	0.125
ALMM	D	0.636	0.000	0.182	0.182	0.000	0.000
AMP1	A	0.000	0.000	0.500	0.000	0.000	0.500
AMP9	B	0.000	0.429	0.143	0.000	0.143	0.286
BAC1	D	0.600	0.000	0.200	0.200	0.000	0.000
BAT1	C	0.167	0.333	0.500	0.000	0.000	0.000
BOTC	D	0.556	0.000	0.333	0.111	0.000	0.000
CARR1	C	0.000	0.111	0.333	0.000	0.333	0.222
CAS2	B	0.000	0.500	0.000	0.000	0.000	0.500
CCD2	C	0.286	0.000	0.571	0.143	0.000	0.000

CCD3	C	0.143	0.000	0.571	0.143	0.143	0.000
CDI	D	0.800	0.000	0.200	0.000	0.000	0.000
CRE14	C	0.200	0.100	0.400	0.200	0.100	0.000
CRE15	C	0.182	0.091	0.545	0.091	0.091	0.000
CRE2	C	0.000	0.125	0.625	0.250	0.000	0.000
CRE22	C	0.143	0.143	0.429	0.143	0.000	0.143
CRE3	C	0.000	0.143	0.571	0.000	0.143	0.143
CRE4B	C	0.125	0.125	0.500	0.000	0.125	0.125
CRE5A	C	0.333	0.000	0.500	0.167	0.000	0.000
CRE6	D	0.444	0.000	0.333	0.111	0.111	0.000
CRE8	C	0.000	0.125	0.625	0.000	0.125	0.125
DHS16	D	0.556	0.000	0.222	0.222	0.000	0.000
ESBA	D	0.500	0.000	0.200	0.300	0.000	0.000
GLO10	C	0.000	0.286	0.714	0.000	0.000	0.000
GLO5	D	0.300	0.000	0.200	0.400	0.000	0.100
HIJ1	B	0.000	0.200	0.400	0.000	0.000	0.400
HORN	C	0.400	0.000	0.400	0.000	0.200	0.000
JUN 2	C	0.000	0.000	0.400	0.200	0.200	0.200
KSS	D	0.200	0.000	0.200	0.600	0.000	0.000
KSSS	D	0.286	0.000	0.286	0.429	0.000	0.000
LIBR	D	0.429	0.000	0.143	0.429	0.000	0.000
LMA	C	0.222	0.111	0.444	0.222	0.000	0.000
LP5H	A	0.000	0.000	0.143	0.000	0.143	0.714
LUP	A	0.000	0.111	0.111	0.000	0.111	0.667
MB2A	C	0.000	0.200	0.600	0.000	0.200	0.000
MB2B	C	0.111	0.111	0.444	0.000	0.222	0.111
MDV2	C	0.200	0.100	0.600	0.100	0.000	0.000
MDV5	C	0.222	0.111	0.556	0.111	0.000	0.000
MDV7	D	0.286	0.000	0.286	0.429	0.000	0.000
MIN1C	D	0.400	0.000	0.200	0.400	0.000	0.000

MOL	A	0.000	0.000	0.222	0.000	0.333	0.444
NGR1	D	0.600	0.000	0.200	0.200	0.000	0.000
NOM	A	0.000	0.125	0.125	0.000	0.250	0.500
NOM10	A	0.000	0.000	0.200	0.000	0.400	0.400
NOM2	C	0.000	0.091	0.364	0.000	0.273	0.273
NOM3	A	0.000	0.000	0.222	0.000	0.444	0.333
NOM4	C	0.000	0.000	0.500	0.000	0.250	0.250
NOM9	B	0.000	0.200	0.200	0.000	0.400	0.200
PAR3	A	0.000	0.000	0.000	0.000	0.167	0.833
PE2A	B	0.111	0.333	0.111	0.000	0.333	0.111
PE2C	B	0.000	0.286	0.143	0.000	0.429	0.143
PER5	C	0.000	0.143	0.429	0.000	0.286	0.143
PERC	C	0.250	0.000	0.750	0.000	0.000	0.000
PERD	C	0.000	0.143	0.714	0.000	0.143	0.000
PIMO	D	0.500	0.000	0.333	0.167	0.000	0.000
PMIN	C	0.111	0.333	0.444	0.000	0.000	0.111
PUR23	D	0.286	0.000	0.286	0.429	0.000	0.000
PUR24A	D	0.333	0.111	0.222	0.222	0.111	0.000
PUR25	D	0.286	0.143	0.286	0.286	0.000	0.000
PUR3	D	0.500	0.000	0.167	0.333	0.000	0.000
REG2	C	0.000	0.000	1.000	0.000	0.000	0.000
ROM11	C	0.167	0.167	0.500	0.000	0.167	0.000
ROM7	C	0.000	0.167	0.500	0.000	0.333	0.000
SALO	D	0.769	0.000	0.154	0.077	0.000	0.000
SIM2	A	0.000	0.125	0.125	0.000	0.000	0.750
SOL	A	0.083	0.083	0.083	0.000	0.333	0.417
TM1	B	0.000	0.333	0.333	0.000	0.000	0.333
TM3	B	0.000	0.600	0.000	0.000	0.200	0.200
TM4	B	0.000	0.750	0.000	0.000	0.000	0.250
TM5	B	0.000	0.500	0.000	0.000	0.000	0.500

TOR1	A	0.000	0.000	0.100	0.000	0.300	0.600
TOR2	A	0.000	0.000	0.143	0.000	0.286	0.571
TOR3A	A	0.000	0.077	0.154	0.000	0.385	0.385
TOR3B	A	0.000	0.000	0.143	0.000	0.429	0.429
TOT	C	0.200	0.000	0.600	0.200	0.000	0.000
TOTA	C	0.125	0.250	0.625	0.000	0.000	0.000
TOTD	C	0.000	0.000	0.800	0.200	0.000	0.000
VDC3	D	0.125	0.000	0.250	0.625	0.000	0.000
VDC6	D	0.111	0.000	0.444	0.444	0.000	0.000
VDM	D	0.300	0.100	0.200	0.400	0.000	0.000
VFEL9	A	0.000	0.143	0.143	0.000	0.286	0.429
VIB22C	C	0.200	0.000	0.600	0.200	0.000	0.000
VILL	D	0.111	0.111	0.222	0.556	0.000	0.000
VIP	C	0.000	0.250	0.625	0.125	0.000	0.000

References for Supplementary text and figures

1 Gómez Cano, A. R., Cantalapiedra, J. L., Álvarez Sierra, M. Á. & Hernández Fernández, M. A macroecological glance at the structure of late Miocene rodent assemblages from Southwest Europe. *Sci. Rep.* **4**, 1-6 (2014).

2 Casanovas-Vilar, I. & Agusti, J. Ecogeographical stability and climate forcing in the Late Miocene (Vallesian) rodent record of Spain. *Palaeogeogr. Palaeoclimatol. Palaeoecol.* **248**, 169-189 (2007).

3 Munger, J. C. & Brown, J. H. Competition in desert rodents: an experiment with semipermeable exclosures. *Science* **211**, 510-512 (1981).

4 Abramsky, Z. & Pinshow, B. The effect of competition on foraging activity in desert rodents: Theory and experiments. *Ecology* **71**, 844-854 (1990).

5 Valone, T. J. & Brown, J. H. Effects of competition, colonization, and extinction on rodent species diversity. *Science* **267**, 880 (1995).

6 Morris, D. W., Fox, B. J., Luo, J. & Monamy, V. Habitat-dependent competition and the coexistence of Australian heathland rodents. *Oikos* **91**, 294-306 (2000).

7 Brown, J. H., Kelt, D. A. & Fox, B. J. Assembly rules and competition in desert rodents. *Am. Nat.* **160**, 815-818 (2002).

8 Domingo, L. *et al.* Late Neogene and early Quaternary paleoenvironmental and paleoclimatic conditions in southwestern Europe: isotopic analyses on mammalian taxa. *PLoS. ONE* **8**, e63739 (2013).

9 García-Alix, A., Minwer-Barakat, R., Suárez, E. M., Freudenthal, M. & Martín, J. M. Late Miocene–Early Pliocene climatic evolution of the Granada Basin (southern Spain) deduced from the paleoecology of the micromammal associations. *Palaeogeogr. Palaeoclimatol. Palaeoecol.* **265**, 214-225 (2008).

10 Suata-Alpaslan, F. The paleoecology of the continental early Pliocene of the eastern Mediterranean, a construction based on rodents. *Fen Bilimleri Dergisi* **31**, 29-48 (2010).

11 Gómez Cano, A. R. *Análisis Bioclimático y Paleoecológico de las Faunas de Roedores del Mioceno Superior Ibérico*, Universidad Autónoma de Madrid, (2013).

12 Gómez Cano, A. R. *et al.* Ecomorphological characterization of murines and non-arvicoline cricetids (Rodentia) from south-western Europe since the latest Middle Miocene to the Mio-Pliocene boundary (MN 7/8–MN13). *PeerJ* **5**, e3646 (2017).

13 Colombero, S. *et al.* Late Messinian mollusks and vertebrates from Moncucco Torinese, north-western Italy. Paleoecological and paleoclimatological implications. *Palaeontol. Electron* **20**, 1-66 (2017).

14 Daams, R. & de Bruijn, H. A classification of the Gliridae (Rodentia) on the basis of dental morphology. *Hystrix, the Italian Journal of Mammalogy* **6**, 3-50 (1995).

15 Daams, R. & Van der Meulen, A. Paleoenvironmental and paleoclimatic interpretation of micromammal faunal successions in the upper Oligocene and Miocene of north central Spain. *Paleobiol. Cont* **14**, 241-257 (1984).

16 Daams, R., Freudenthal, M. & Van der Meulen, A. Ecostratigraphy of micromammal faunas from the Neogene of Spain. *Scripta Geologica, Special Issue* **1**, 287-302 (1988).

17 van Dam, J. A. & Weltje, G. J. Reconstruction of the Late Miocene climate of Spain using rodent palaeocommunity successions: an application of end-member modelling. *Palaeogeogr. Palaeoclimatol. Palaeoecol.* **151**, 267-305 (1999).

18 de Bruijn, H. & Ünay, E. On the evolutionary history of the Cricetodontini from Europe and Asia Minor and its bearing on the reconstruction of migrations and the continental biotope during the Neogene. *The Evolution of Western Eurasian Neogene Mammal Faunas. Columbia University Press, New York*, 227-234 (1996).

19 de Bruijn, H. in *The Miocene Land Mammals of Europe.* (eds E. Rössner & K. Heissig) 271-280 (Verlag Dr. Friedrich Pfeil, 1999).

20. Fejfar, O. Microtoid cricetids. *The Miocene land mammals of Europe*, 356-371 (1999).
21. Freudenthal, M. & Mein, P. *Description of Fahlbuschia (Cricetidae) from various fissure fillings near La Grive-St. Alban (Isère, France)*. (Rijksmuseum van Geologie en Mineralogie, 1989).
22. Hernández Fernández, M. & Peláez-Campomanes, P. Ecomorphological characterization of Murinae and hypsodont" Cricetidae"(Rodentia) from the Iberian Plio-Pleistocene. *Coloquios de Paleontología*, 237-251 (2003).
23. Kälin, D. Tribe cricetini. *The Miocene land mammals of Europe*, 373-387 (1999).
24. Martín-Suárez, E., Freudenthal, M. & Civis, J. Rodent palaeoecology of the continental upper Miocene of Crevillente (Alicante, SE Spain). *Palaeogeogr. Palaeoclimatol. Palaeoecol.* **165**, 349-356 (2001).
25. Mein, P. Les sciuropteres (Mammalia, Rodentia) neogenes d'Europe occidentale. *Geobios* **3**, 7-77 (1970).
26. Rummel, M. Tribe cricetodontini. *The Miocene Land Mammals of Europe. Verlag Dr. Friedrich Pfeil, München*, 359-364 (1999).
27. Wessels, W. in *The Miocene Land Mammals of Europe* (eds G.E. Rössner & K. Heissig) 301-318 (1999).
